# Microbiome and Functional Analysis of a Traditional Food Process: Isolation of a Novel Species (*Vibrio hibernica*) With Industrial Potential

**DOI:** 10.3389/fmicb.2020.00647

**Published:** 2020-04-09

**Authors:** David F. Woods, Iwona M. Kozak, Fergal O’Gara

**Affiliations:** ^1^BIOMERIT Research Centre, School of Microbiology, University College Cork, Cork, Ireland; ^2^Human Microbiome Programme, School of Biomedical Sciences, Curtin Health Innovation Research Institute, Curtin University, Perth, WA, Australia; ^3^Telethon Kids Institute, Perth Children’s Hospital, Perth, WA, Australia

**Keywords:** brine, Wiltshire, genome, *Vibrio*, next generation sequencing, phylogeny, bioinformatics, food

## Abstract

Traditional food preservation processes are vital for the food industry. They not only preserve a high-quality protein and nutrient source but can also provide important value-added organoleptic properties. The Wiltshire process is a traditional food curing method applied to meat, and special recognition is given to the maintenance of a live rich microflora within the curing brine. We have previously analyzed a curing brine from this traditional meat process and characterized a unique microbial core signature. The characteristic microbial community is actively maintained and includes the genera, *Marinilactibacillus, Carnobacterium, Leuconostoc*, and *Vibrio*. The bacteria present are vital for Wiltshire curing compliance. However, the exact function of this microflora is largely unknown. A microbiome profiling of three curing brines was conducted and investigated for functional traits by the robust bioinformatic tool, Tax4Fun. The key objective was to uncover putative metabolic functions associated with the live brine and to identify changes over time. The functional bioinformatic analysis revealed metabolic enrichments over time, with many of the pathways identified as being involved in organoleptic development. The core bacteria present in the brine are Lactic Acid Bacteria (LAB), with the exception of the *Vibrio* genus. LAB are known for their positive contribution to food processing, however, little work has been conducted on the use of *Vibrio* species for beneficial processes. The *Vibrio* genome was sequenced by Illumina MiSeq technologies and annotated in RAST. A phylogenetic reconstruction was completed using both the 16S rRNA gene and housekeeping genes, *gapA*, *ftsZ*, *mreB*, *topA*, *gyrB*, *pyrH*, *recA*, and *rpoA*. The isolated *Vibrio* species was defined as a unique novel species, named *Vibrio hibernica* strain B1.19. Metabolic profiling revealed that the bacterium has a unique substrate scope in comparison to other closely related *Vibrio* species tested. The possible function and industrial potential of the strain was investigated using carbohydrate metabolizing profiling under food processing relevant conditions. *Vibrio hibernica* is capable of metabolizing a unique carbohydrate profile at low temperatures. This characteristic provides new application options for use in the industrial food sector, as well as highlighting the key role of this bacterium in the Wiltshire curing process.

## Introduction

The use of microorganisms in processing technologies has a rich and sophisticated narrative throughout history. This ranges from ancient textile techniques, to the production of antimicrobials, sewage treatment and utilizing biotechnology in the construction industry for microbially mediated construction processes and biomaterial production (Fleming, 2001; Dhall et al., 2012; Liu et al., 2014; Stabnikov et al., 2015; Dapurkar and Telang, 2017; Heine et al., 2019). Microbial processing technologies have also been widely implemented in the food industry. Our increased knowledge of microbial processing of food has led to the incorporation and encouragement of specific microbial activities to form a safer, healthier and more flavorsome end products (Singh et al., 2016). These microbiological processes can produce a value-added product in comparison to the raw material inputs. Bacteria have been used in the production of yogurt, in the >1000 varieties of cheese, olives, kefir, wines, kimchi and in sausage meat to name but a few (Sandine and Elliker, 1970; Lücke, 2000; Fleet, 2008; Kearney et al., 2011; Jung et al., 2012; Gul et al., 2015; Papadelli et al., 2015; Fontana et al., 2016; De Pasquale et al., 2019).

Fermented meat products are of particular importance with regards to the maintenance of their nutrients as well as the increase in organoleptic qualities and market value (Fontana et al., 2016). Research into the microbial composition of traditional food processes is a rapidly growing area with particular emphasis put on the genomic potential of microorganisms present within a process. However, the complex dynamics mediated by the microbial milieu during food processing require further investigation and this is particularly evident with traditional meat processes (Bokulich et al., 2012b, 2016). There is a strong need to investigate the overall function of these processes as well as their component parts, to elucidate the activities that the microbes are completing. The definition of normal and abnormal microbial compositions is vital at every stage of bacterial mediated food processing. This goal is essential to understand key issues underpinning organoleptic consistency, problem diagnostics, spoilage investigation and process improvement (Bokulich et al., 2012a, 2016).

Both culture-dependent and culture-independent based techniques have been used to examine the microflora present in meat. Culture-dependent techniques are not commonly applicable to many food-associated niches for complete microbial characterization. This is due to difficulties in designing and reproducing the appropriate growth media and conditions to enable indigenous microbes to grow under laboratory conditions (Amann et al., 1995). However, the culturing of microbes is an important step to gain more insights into bacterial function and for the potential development of inocula (Hugas and Monfort, 1997). With regards to culture-independent techniques Next Generation Sequencing (NGS) technologies have advanced rapidly and have been applied to solve the difficulties of obtaining accurate microbial profiles. The rate of NGS technological advancements has been noted as being significantly higher than the rate of standard computational power advancements based on Moore’s Law (Batt, 2016). The NGS of marker genes, such as the 16S rRNA gene, provides an accurate snapshot of the bacterial content within a food process and has been successfully applied to characterize a number of traditional meat processes (Mayo et al., 2014; Franciosa et al., 2018; Woods et al., 2019). Moreover, NGS sequence datasets could be applied to functional prediction algorithms such as Tax4Fun to aid in the interrogation of the potential metabolic capabilities of traditional bacterial food processes (Aßhauer et al., 2015). Thus, giving a putative overview of a process at a particular time. While Tax4Fun has been predominantly utilized for disease prediction and dysbiosis, traditional food practices can be very intricate and this functional prediction software could illuminate the processing capabilities of the bacteria present (Coffey et al., 2019; Sun et al., 2019; Wang et al., 2019).

Wiltshire curing is a traditional process which yields high-quality cured hams. Wiltshire curing is seen as both culturally and economically important, and there is special recognition given to the maintenance of the bacterial component of the process (Andersen, 2004). Meat is cured in microbially rich brine for 5–7 days at a temperature of 4°C. After approximately 40 days use, brine that is deemed to be spent is replenish with half fresh brine to maintain the microbial component (Andersen, 2004). Curing/processing of meat at lower temperature decreases the propagation of most pathogenic bacteria, which are predominantly mesophilic (Húngaro et al., 2014). Using cold adapted bacteria, referred to as psychrophiles, to process food at lower temperatures would be advantageous and increase the food safety of a product (Durán Quintana et al., 1999). This Wiltshire process has been successfully profiled for its core microbial content (Woods et al., 2019). The core genera identified in a Wiltshire compliant brine were *Marinilactibacillus, Carnobacterium, Leuconostoc*, and *Vibrio*. Species of *Marinilactibacillus, Carnobacterium*, and *Leuconostoc* are known as lactic acid producing bacteria and these Lactic Acid Bacteria (LAB) strains are often used in food processing (Toffin et al., 2005; Lucena-Padros et al., 2015; Pogacic et al., 2016; Soto Del Rio Mde et al., 2016; Liu et al., 2017; Saraoui et al., 2017). A *Vibrio* species has previously been identified in a Wiltshire compliant brine with no detrimental effect to the end product (Hinrichsen et al., 1994; Woods et al., 2019). Moreover, an unidentified autochthonous *Vibrio* species from a Wiltshire brine, was used to inoculate a brine and the meat was cured in this mixture. The *Vibrio* was shown to improve the organoleptic qualities of the meat specifically with regards to the color and flavor of the cured meat (Petaja et al., 1972).

The genus of *Vibrio* is one of the most diverse bacterial genera and has more than 142 species defined (Sawabe et al., 2013 2014). *Vibrio* species are ubiquitous in marine, estuarine and freshwater environments, and have been isolated from a wide variety of organisms including fish, plankton and mammals (Thompson et al., 2004; Reen et al., 2006; Froelich et al., 2013). The genus consists of Gram-negative short rod-shaped halophilic bacteria. The genome of *Vibrio* is organized into a two-chromosome configuration. The larger chromosome is predominantly between 3.0 and 3.3 Mb and the smaller between 0.8 and 2.4 Mb (Okada et al., 2005).

While much research has been conducted on the pathogenicity and the detrimental cost of notorious *Vibrio* species such as *V. cholerae* and *V. parahaemolyticus*, there is a plethora of non-pathogenic *Vibrio* species which harbor beneficial and industrially important applications (Makino et al., 2003; Bertuzzo et al., 2010). *V. natriegens* is an example of an industrially relevant bacteria which has been recognized as a novel candidate for future biotechnological processes due to its high biomass specific consumption rate in industrial fermentation processes (Hoffart et al., 2017). The marine *V. costicola* has important pharmaceutical relevance, as it is capable of producing the potent antileukemic agent L-Glutaminase and shows great substrate specificity over a wide variety of conditions (Nagendra Prabhu and Chandrasekaran, 1997). The genus has also been recognized for its importance in aquaculture. Investigation into the gut microbiota of healthy fish has seen the predominance of *Vibrio* species in a wide spectrum of studies, thus potentially identifying *Vibrio* as a key component of the healthy fish gut microbiome (Egerton et al., 2018).

In order to better understand the contributing effects of the microbial components of a Wiltshire cure, our study investigated the functional possibilities of the microbial content. We first predicted the potential functions of the microbes present in the brine, and at the different time points. We subsequently focused our study on the only non-LAB genus identified as a major component of the core microbiome. To assess the possible contribution of the *Vibrio* species to the overall organoleptic qualities of traditionally cured Wiltshire compliant products, we sequenced and studied the genome. The overall microbial functional analysis provided an insight into the prospects and opportunities of the total brine. In contrast, the genome was investigated for functionality with regards to meat processing and improvements to organoleptic related food properties. We investigated the metabolism of this *Vibrio* species and showed a wide range of metabolic capabilities that could be industrially relevant and are related to flavor development. Moreover, the bacteria are able to metabolize industrially relevant molecules at 4°C. Therefore, the investigation into the capabilities of the bacterial genome is vital to further advance food processing and to increase our insight into the complex matter of flavor development.

## Materials and Methods

### Sampling and Characterization of an Active Wiltshire Brine

To sample and characterize the brine, procedures were followed from Woods et al. (2019). Brine was sampled from a fully Wiltshire compliant facility longitudinally at three timepoints, Day 0 where the brine is composed of half “spent” brine and half newly reconstituted brine, Day 20, where this brine was used for 20 days to cure different meats and Day 40, where the brine was deemed to be “spent.” The brine was sampled in late 2016 from three monitored curing containers. These samples are distinct from the previously characterized brine samples in Woods et al. (2019). To culture the brine microflora, Tryptic Soy Broth (TSB) (Merck) with 1.5% Agar (Merck) (TSA) ± additional 6% NaCl (Sigma) was used, incubated at 23°C for 2 weeks. Unique morphotypes were isolated and stored at −80°C.

To characterize the microbiome of the brine, 1 ml of brine was centrifuged at 13,000 × *g* for 5 min and the total genomic (g)DNA isolated using the Puregene DNA Extraction kit (QIAGEN). The quantity and quality were assessed by a Qubit 2.0 Fluorometer (Life Technologies) and a 0.5% agarose gel (Sigma), respectively. MWG Eurofins (Ebersberg, Germany) conducted the microbiome analysis from the isolated gDNA, by a tagged 16S rRNA gene PCR of the v3-v5 region and sequenced the amplicons on a Next Gen Illumina MiSeq (V3), 2 × 300 bp. Raw reads were demultiplexed and sequences were assigned an Operational Taxonomic Unit (OTU) in QIIME 1.9.0 (Caporaso et al., 2010). Bar charts were created in Excel. The raw reads are publicly available in the Sequence Read Archive (SRA) of the NCBI Database under Accession numbers; SRR11073122, SRR11073123, SRR11073124, SRR11073125, SRR11073126, SRR11073127, SRR11073128, SRR11073129, SRR11073130, SRR11073131, SRR11073132, SRR11073133, SRR11073134, SRR11073135, SRR11073136, SRR11073137, SRR11073138, SRR11073139, SRR11073140, SRR11073141, SRR11073142, SRR11073143, SRR11073144, SRR11073145, SRR11073146, SRR11073147, and SRR11073148.

### Functional Bioinformatic Analysis of a Wiltshire Brine (Tax4Fun)

R package Tax4Fun was used to predict the possible functional capabilities of the microbial communities in the analyzed brine samples (Aßhauer et al., 2015). R version 3.3.2 was used to host the Tax4Fun package. The biom file of the microbiome data of each of the brine samples profiled in this study was converted to a text file in QIIME 1.9.0 (Caporaso et al., 2010) and imported to Tax4Fun (importQIIMEData). The SILVA123 database was downloaded from the Tax4Fun website^1^ and used to predict functional capabilities. The Tax4Fun result lists were formatted into data frames and saved as Comma-Separated Values (csv) files. This was imported into Microsoft Excel where the data was delineated by a comma. To produce the heatmap, the three technical replicates were averaged and each individual KEGG of the biological replicates was conditionally formatted to a color scale. All relevant data was included when above the overall average of all of the dataset. The data were arranged by the 0_1 sample set for clarity and trend identification. Within sample replicate variance was calculated in Excel.

### Amplification of the 16S rRNA Gene for Species Identification

Morphotypes were identified by amplifying the 16S rRNA gene, with the previously published primer set, forward primer 63f (5′-CAG GCC TAA CAC ATG CAA GTC-3′) and reverse primer 1387r (5′-GGG CGG WGT GTA CAA GGC-3′) using the Q5 Hot Start High-Fidelity Polymerase system (New England Biolabs) (Marchesi et al., 1998). The following thermocycler conditions were used, initial activation at 98°C for 30 s and then subjected to 35 cycles of amplification (denaturation at 98°C for 10 s, annealing at 58°C for 30 s and elongation at 72°C for 90 s) followed by a final elongation step at 72°C for 2 min (BIOMETRA T3000). Amplicons were visualized on 0.8% agarose gels stained with SYBR Safe (Thermo Fisher Scientific) and sequenced by Eurofins Genomics^2^. Consensus sequences were achieved using Unipro UGENE version 1.27 and species identification was assessed using the *in silico* BLAST (NCBI) algorithm (Altschul et al., 1990; Okonechnikov et al., 2012).

### Genome Extraction and Sequencing

Genomic DNA (gDNA) was isolated using the Gentra Puregene DNA Isolation Kit (Qiagen). The gDNA was checked for quantity using a Qubit 2.0 Fluorometer (Life Technologies) and the integrity assessed on a 0.5% agarose gel (Sigma). Library preparation, validation and sequencing was conducted by Eurofins Genetic Services. In brief, the genome was sequenced using the Illumina MiSeq platform with chemistry v3 and 2 × 300 bp paired-end sequencing.

### Genome Assembly and Annotation

The assembly of the genome was conducted using Unicycler (Galaxy version 0.4.6.0) for Illumina short reads in the FASTQ format to produce complete and accurate assemblies (Wick et al., 2016). Contigs shorter than 1,000 bp were excluded from the final assembly. Genes were predicted and annotated using the Rapid Annotation Subsystem Technology (RAST version 2.0) software with the Classic RAST algorithm (Aziz et al., 2008; Overbeek et al., 2014). The BLAST function was used for additional annotation of genes.

### Bioinformatic Species Definition

Two *in silico* methods of species definition were used to define *Vibrio hibernica* strain B1.19 as a novel species. The reference genomes of the following *Vibrio* species were downloaded in FASTA format from the National Centre for Biotechnology Information (NCBI) website: *V. rumoiensis* (NZ_AP018685.1, NZ_AP018686.1, NZ_AP018687.1, NZ_AP018688.1), *V. litoralis* (NZ_AUFZ00000000.1), *Vibrio scophthalmi* (NZ_CP016307.1, NZ_CP016308.1, NZ_CP016309.1, NZ_CP016310.1NZ_CP016311.1), *Aliivibrio fischeri* (NC_006840.2, NC_006841.2, NC_006842.1) and *V. parahaemolyticus* (NC_004603.1, NC_004605.1). The assembled genome of *V. hibernica* in FASTA format was uploaded to the ANI Calculator^3^ and compared to the reference genomes of other *Vibrio* species. The other method used in this study was *in silico* DNA-DNA Hybridization (DDH) using the Genome-To-Genome Distance Calculator (GGDC). The *V. hibernica* and reference genomes of other *Vibrio* species tested were uploaded in a pair-wise comparison to the GGDC website^4^.

### Molecular Phylogenetic Analysis

To infer the phylogenetic history, nucleotide sequences were retrieved and downloaded from NCBI: GenBank^5^. The House Keeping (HK) genes used for the phylogenetic reconstruction were *ftsZ*, *gapA*, *gyrB*, *mreB*, *pyrH*, *recA*, *rpoA*, and *topA*. Sequence alignments were generated for the HK and 16S rRNA trees using Clustal Omega and trimmed to a consistent length (Sievers et al., 2011). The HK alignments were concatenated into a single alignment. The evolutionary analysis was conducted in MEGA X via the Maximum Likelihood (ML) method using the General Time Reversible model for the HK tree and the Kimura 2-parameter model for the 16S rRNA tree (Kimura, 1980; Nei and Kumar, 2000; Kumar et al., 2018). A discrete Gamma distribution with Invariant (G + I) sites was used to model evolutionary rate differences among sites. The phylogenetic distribution was tested by the Bootstrapping method using 1,000 replicates (Felsenstein, 1985). Nucleotide sequences from *Salinivibrio costicola*, *Enterovibrio coralii*, and *Photobacterium lutimaris* were used as Outgroups for the phylogenetic reconstruction. Details on the HK and 16S rRNA sequences accession numbers are in Supplementary Tables S1, S2.

### Phenotypic Analysis of *Vibrio hibernica*

The strains used for comparative analysis included *V. hibernica* sp. nova (Vh) strain B1.19, *V. litoralis* DSM17657 (Vl), *A. fischeri* MJ11 (Af), *V. scophthalmi* DSM19140 (Vs), *V. parahaemolyticus* RIMD 2210633 (Vp) and *V. gallaecicus* DSM23502 (Vg). The temperature growth range was assessed on TSB and TSA (Merck), Marine Agar (MA) (BD Difco), Brain Heart Infusion (BHI) (Merck) and Nutrient Agar with NaCl (NA) (Peptone (Merck) 5 g/l, Meat Extract (Sigma) 3 g/l and Agar (Sigma) 15g/l at 4°C, 23°C, 30°C, and 37°C. Single colonies were tested for catalase activity with 10% hydrogen peroxide (Sigma) and cultured on Columbia Blood Agar (CBA) (Fannin) at 23°C for 48 h to assay hemolysis. Salt tolerance was conducted in TSB (Merck) supplemented with 0, 3, 6, 10, and 20% additional NaCl (Sigma) and grown at 23°C with agitation (180 rpm) for 24 h. To assess the biochemical profile of the organisms, an API 20NE kit (BioMerieux) was used with API NaCl Medium (BioMerieux). To profile the carbohydrate metabolism range of the bacteria, API 50CH kits (BioMerieux) were used in conjunction with API 50 CHB/E Medium (BioMerieux). Both the API 20NE and API 50CH were used with manufacturers guidelines with the exception of the temperatures used, 4 and 23°C. All assays were completed with three independent biological replicates.

## Results

### Microbial Profile and Functional Prediction of an Active Wiltshire Cure

The traditional Wiltshire curing process was profiled to determine the microbial signature of an active curing process. The brine was sampled in late 2016 from three monitored curing containers, Day 0, at the initial replenishing phase, where half of the spent brine is replaced with half freshly reconstituted brine, Day 20, where the brine had been used for 20 days and Day 40 where the brine was used for 40 days and thus designated as needing to be replenished to start the cycle anew. Samples were taken in triplicate for each sampling point. The microbial constituents of the meat cure were profiled by microbiome analysis. *Marinilactibacillus, Carnobacterium, Leuconostoc*, *Vibrio*, and *Photobacterium* were the dominant bacteria present. To further assess the consistency and stability of the Wiltshire microflora, a longitudinal microbiome profile of brine from three meat processing containers was conducted. Samples were taken at three time points, with three sampling replicates included. We compared this microbiome, sampled in late 2016, to Wiltshire brine microbiomes that we conducted in early 2016 and in 2014 and this demonstrated a highly similar profile in each (Supplementary Figure S1). Again, the profile signature was dominated with the genera, *Marinilactibacillus, Carnobacterium, Leuconostoc*, and *Vibrio*. The Genus *Photobacterium* was not present as a dominant member in all of the previous studies (Woods et al., 2019). Hence this Genus was not included as a stable dominant microbial member of the Wiltshire brine signature. There was minor variation within the sampling replicates and the microbial diversity percentages between brine samples, however, overall the profiles are highly similar (Supplementary Figure S1).

**FIGURE 1:**
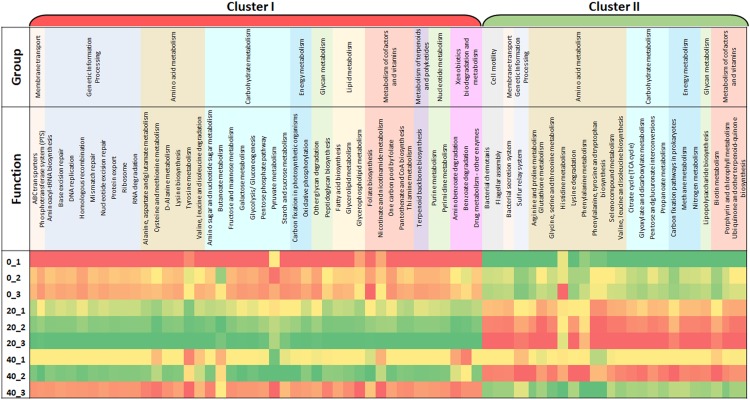
This heat map illustrates changes in functional capabilities of a brine microbiome at three timepoints, immediately after the initial replenishment of the Wiltshire brine (Day 0, denoted _0), during the use of the brine (Day 20) and just before the spent brine is replenished (Day 40). At each timepoint three replicates of brine was sampled to give an average value. Three containers of brine were monitored in tandem Container 1, 2, and 3. Samples are designated as Container Number_Day, for example 0_1 is equivalent to Day 0_Container 1. The data sets cluster into two major sets, Cluster I and II. Cluster I shows a functional enrichment increase from Day 0 to Day 20 and are subsequently selectively decreased for by Day 40, and Cluster II shows a decreased enrichment from Day 0 until Day 20 and selectivity enriched for by Day 40. The color scale ranges from red through yellow and to green, red indicates a low functional enrichment, yellow, a medium functional enrichment and green, a high functional enrichment of the averaged individual KEGG function.

To further improve our understanding of this food curing process, it was necessary to analyze the function of the microbes within the brine samples. Using the powerful bioinformatic tool Tax4Fun, a functional prediction of the microbial capabilities of a Wiltshire brine was analyzed over time (Figure 1). The three individual cures that were sampled at three time points show similar trends from Day 0 to Day 20 up until Day 40. Within the triplicate samples for Day 0 and Day 20 a similar profile was observed, whereas Day 40 samples showed greater variability when compared. There are two major categories of functional groups, Cluster I where functional enrichment increases from Day 0 to Day 20 and subsequently shows a decrease by Day 40. Within Cluster II, there is a decreased enrichment from Day 0 until Day 20 and an increase in enrichment by Day 40. Metabolic and cellular functions were placed in an overall grouping such as amino acid and carbohydrate metabolism. Within Cluster I there are amino acid metabolism systems such as valine, leucine and isoleucine degradation, D-alanine metabolism, lysine biosynthesis, cysteine and methionine metabolism, alanine, aspartate, and glutamate metabolism. There are also amino acid metabolism systems in Cluster II such as phenylalanine metabolism, valine, leucine, and isoleucine biosynthesis, lysine degradation, phenylalanine, tyrosine and tryptophan biosynthesis, glycine, serine and threonine metabolism and arginine and proline metabolism. There are a number of genes annotated to functions involved in the metabolism of complex carbohydrates, most of these are in Cluster I. These included galactose metabolism, other glycan degradation, the pentose phosphate pathway, glycolysis/gluconeogenesis, starch and sucrose metabolism, amino sugar and nucleotide sugar metabolism. While genetic functions such as the citrate acid cycle (TCA), glyoxylate and dicarboxylate metabolism and glutathione metabolism showed an enrichment in Cluster II. Both functions for ABC transporters and folate biosynthesis are present in Cluster I and show an increase enrichment from Day 0 to Day 20. This analysis provides a functional snapshot of a Wiltshire brine immediately after replenishment (Day 0), during full productive use (Day 20) and toward a spent brine phase (Day 40). It is likely that the functions most important for Wiltshire curing are those that are enriched for in Cluster I on Day 20, thus revealing a predicted functional profile for a fully compliant curing process.

### Genomic Components and Characteristics of the *Vibrio* Strain Isolated From Wiltshire Brine

Initially to investigate the novelty of the *Vibrio* isolate (hereto named *Vibrio hibernica*), we sequenced the 16S rRNA marker gene. We analyzed ten independently cultured isolates, randomly selected *Vibrio* morphotypes from different brine containers on different days. The 16S rRNA marker gene was sequenced in each and showed sequence homology supported by at least three independent reads of each nucleotide. This analysis showed 98% sequence identity with the closest published *Vibrio* species, *V. litoralis* (DQ097524.1). The phylogenetic analysis of members of the *Vibrio* genus was reconstructed using the common genetic marker of the 16S rRNA gene (Supplementary Figure S2). The 16S rRNA gene tree was not supported well with the 1,000 bootstraps. However, this tree gave us an indication of the closely related species (*V. litoralis*) and *Vibrio* species from different clades (*V. parahaemolyticus*, *V. scophthalmi, A. fischeri*, and *V. gallaecicus*).

**FIGURE 2:**
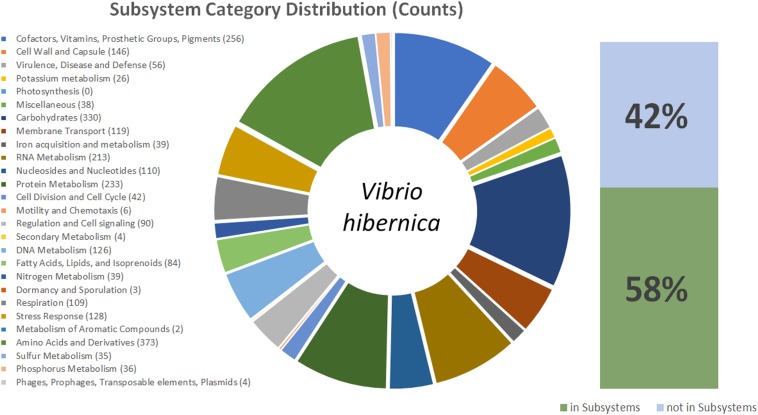
This figure shows a graphical representation of the *V. hibernica* genome. Annotation was completed using the ClassicRAST annotation scheme. The Subsystem Categories Distributions are listed and in brackets the number of genes assigned to each category. The pie chart illustrates the distribution of genes among the different categories in *V. hibernica*. Also included are the percentages of the annotated genes of *V. hibernica* found in Subsystems 42% and not in Subsystems 58%.

Due to the 16S rRNA gene having sequence novelty, the genome of *V. hibernica* was sequenced. The genome comprised of 3,425,268 bp with an N50 of >60 kb and an average GC content of 40.6% (Table 1). There is a predicted total of 3,151 protein coding genes within the genome and 57 RNA genes. The data of the genome sequence of *V. hibernica* strain B1.19 that is studied here is deposited in DDBJ/ENA/GenBank under the accession VHKO00000000. The analysis from the RAST SEED Viewer revealed that there are 481 subsystems present in the genome analysis (Figure 2).

**TABLE 1:** General features of the *Vibrio hibernica* genome.

*Vibrio hibernica*	
Genome size (bp)	3,425,268
Contigs	135
Contigs N50 (bp)	60,909
GC content (%)	40.6
Protein coding genes	3,151
RNA genes	57

### Bioinformatic Species Distinction of *Vibrio hibernica*

To further assess and define *V. hibernica* as a species, bioinformatic tools were employed. The ANI algorithm measures the nucleotide level genomic similarities between the coding regions of two genomes. When *V. hibernica* was compared to each of the species tested *V. rumoiensis* and *V. litoralis* were shown to be the closest relative based on sequence similarity at 75.77 and 75.61% respectively (Table 2). Using the *in silico* DNA–DNA Hybridization method to assay species distinction, *V. hibernica* differed from all of the tested genomes by greater than 1%. Again, the genomes of *V. rumoiensis* and *V. litoralis* were the closest with regards the GC% when compared to *V. hibernica*. *V. parahaemolyticus* differed from the Wiltshire brine isolate by the most (>4.05% GC difference) (Table 2).

**TABLE 2:** Genome comparative analysis of *Vibrio hibernica* for species definition against closely related *Vibrio* species.

Species	OrthoANIu (%)	isDDH (± %GC)
*Vibrio rumoiensis*	75.77	1.74
*Vibrio litoralis*	75.61	1.36
*Vibrio scophthalmi*	71.95	4.05
*Aliivibrio fischeri*	71.89	2.22
*Vibrio parahaemolyticus*	71.24	4.80

### Phylogenetic Reconstruction of Species of the Genus *Vibrio*

**FIGURE 3:**
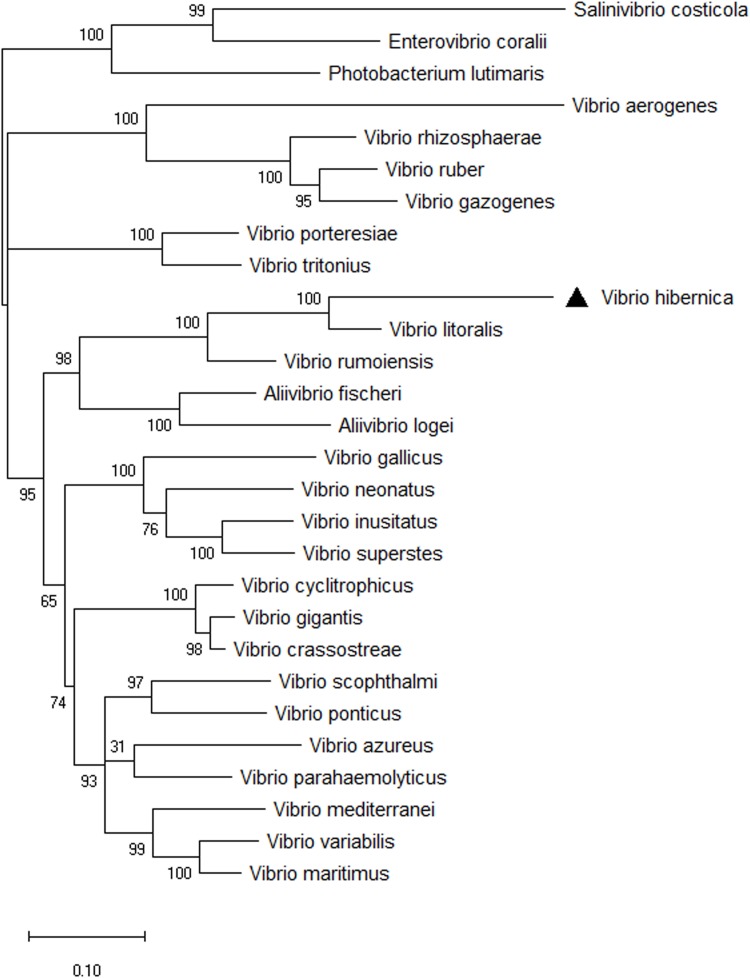
Phylogenetic tree of eight HK genes, *ftsZ, gapA, gyrB, mreB, pyrH, recA, rpoA*, and *topA* from selected *Vibrio* species. The evolutionary history was inferred using the Maximum Likelihood method based on the General Time Reversible model. A discrete Gamma (+G) distribution was used with rate variation model to allow evolutionarily invariable (+I). The phylogeny was tested using 1,000 Bootstrap Replicates. Three outgroups were used to root the three: *Salinivibrio costicola*, *Enterovibrio coralii*, and *Photobacterium lutimaris*. There was a total of 3,778 positions in the final dataset. *V. hibernica* is indicated by a black triangle.

The 16S rRNA phylogenetic tree provided information on the novelty and evolutionary branching of this *Vibrio* isolate. However, using the 16S rRNA gene as a phylogenetic indicator can be problematic and could lead to inaccuracies in generating phylogenetic trees. This can arise from such factors as multiple copies of the gene with variable sequences often being present in the genome (Thompson et al., 2009; Kembel et al., 2012; Louca et al., 2018). To combat this difficulty, a phylogenetic analysis of members of the *Vibrio* genus was reconstructed using eight HK genes (Figure 3). All of the nodes were supported well within the HK tree, with the exception of the discrimination between *V. parahaemolyticus* and *V. azureus*. The outgroups used to root the tree clustered together, and importantly clustered distinctly from all of the *Vibrio* strains present. *V. hibernica* isolated from Wiltshire brine was distinct from the other biological entities present. It grouped with the other *Vibrio* species present, not the outgroups, thus further supporting the bacterial assignment as a member of the genus of *Vibrio*. *V. rumoiensis* and *V. litoralis* grouped on a discrete clade with *V. hibernica*. Both *V. rumoiensis* and *V. litoralis* were previously defined as members of the Rumoiensis clade and we propose that our *V. hibernica* is a member of this clade (Sawabe et al., 2013; Sawabe et al., 2014).

### Functional Genomics of *V. hibernica* to Identify Putative Organoleptic Related Properties

The functional genomic potential of *V. hibernica* was interrogated from its annotated genome to understand the possible role of the microbe in the Wiltshire curing process, as well as any future industrial applications of the strain. Within the genome 58% of the genes were successfully placed in a subsystem (Figure 2). There are 373 genes annotated to be associated with amino acids and their derivatives, this is the largest annotated group within the genome (Figure 2). Within this group there are 12 genes involved in the degradations of methionine. Cysteine, another volatile organic sulfur compound, is a precursor of methionine and there are 20 annotated genes for the biosynthesis of this compound and 7 genes annotated in the production of ribose. The enzymatic machinery for the production of another amino acid, serine, is present in the genome. There are 8 genes present for RNA processing and degradation. Methionine, cysteine, ribose, serine and RNA degradation are all important flavor contributors, especially with regards umami taste (Solms, 1969; Deoda and Singhal, 2003; Gonda et al., 2010). The genome has 15 annotated genes for the biosynthesis of threonine. This is an essential amino acid which cannot be synthesized by humans and must be obtained from our diet.

The subsystem of carbohydrates has the second highest number of annotated features with 330 counts within the genome (Figure 2), with a wide range of sugar metabolism genes, from simple sugars to more complex polysaccharides. The degradation of carbohydrates is one the most important contributors to a food process for improved flavor perception (Weenen and Apeldoorn, 1996). Within this subgroup, central carbohydrate metabolism accounts for most of the features (99), however there are also a number of genes for fermentation (39), monosaccharides (68), di- and oligo-saccharides (31) and aminosugars (13). There are 17 genes responsible for the biosynthesis of heme and siroheme from glycine and L-glutamate. Heme is known for having a pleasing red color and would be specifically beneficial to Wiltshire specific organoleptic traits (Chasco et al., 1996). Genes are present for the biosynthesis of the important osmolyte betaine. There were 56 features identified in the virulence, disease and defense category, these included genes related to resistance to toxic compounds (40) including copper, cobalt, zinc, cadmium, fluoroquinolones, arsenic and tetracycline, as well as efflux pumps. Of the remaining 16 features these included the production of an antibacterial peptide, specifically Colicin and genes involved in invasion and intracellular resistance. One of the most important attributes of the genome is that there are no genes annotated by RAST to be involved in toxin and superantigen production, as well as no genes identified to be present in the category of pathogenicity islands.

There is a large number of genes annotated to be involved in the production of vitamins which are of industrial and market value. Genes (16) are present for the biosynthesis of the essential cofactor vitamin B_1_ (Thiamin). There are 34 genes annotated to be involved in the biosynthesis of vitamin B_7_, commonly known as biotin. The complete gene mechanism needed to convert chorismate to menaquinone (vitamin K_2_), an important vitamin involved in the coagulation of blood, is also present. There are a number of genes annotated for the production of the important coenzyme, ubiquinone, again produced from the compound chorismate. For the biosynthesis of the coenzyme B_12_, 12 genes are present which are involved in the production of this important compound. There are 38 genes and 58 genes involved in the production of folate and pterines, respectively. As well as vitamins there are genes present that are involved in the production of the antioxidant glutathione. These compounds are all categories as value-add ingredients and when available in a food product can provide an enhanced potential for marketability of a product.

### Phenotypic and Biochemical Profiles of *Vibrio hibernica* With Industrial Relevance

Additional metabolic related assays were performed to further characterize *V. hibernica* (Vh) as a species, as well as to give practical examples of industrially relevant processes. The strains phenotypic characteristics were compared and contrasted to closely related species. Both API 20NE and 50CH kits were used to test the practical metabolic capabilities of *V. hibernica* (Vh) and to highlight this isolates distinctiveness. Phenotypically all of the *Vibrio* species tested were oxidase positive and were able to reduce nitrates to nitrite, an important characteristic of a Wiltshire curing microbe (Table 3) (Andersen, 2004). All strains fermented glucose (GLU) with the exception of *V. gallaecicus* (Vg). However, only *V. parahaemolyticus* (Vp) and *V. hibernica* (Vh) were able to metabolize glucose in the presence of oxygen (GLU) and both were able to assimilate malate. *V. gallaecicus* (Vg), *V. parahaemolyticus* (Vp) and *V. hibernica* (Vh) were each able to hydrolase galactosides to monosaccharides. *V. hibernica* (Vh) was able to assimilate *N*-acetyl-glucosamine (NAG) and malate (MLT). The closest phylogenetically related species, *V. litoralis* (Vl), showed a completely different and limited profile compared to the brine isolate, *V. hibernica* (Vh).

**TABLE 3:** Differential biochemical characteristics of examined species of *Vibrio* in this study.

	*Vp*	*Vl*	*Vs*	*Vg*	*Af*	*Vh*
***Test***						
*NO3*	+	+	+	+	+	+
*TRP*	+	−	−	+	−	−
*GLU*	+	+	+	−	+	+
*ADH*	+	−	−	−	−	−
*URE*	+	−	−	−	−	−
*ESC*	−	−	+	+	−	−
*GEL*	+	−	−	−	−	−
*PNG*	+	−	−	+	−	+
*GLU*	+	−	−	−	−	+
*ARA*	+	−	−	−	−	−
*MAN*	+	−	−	−	−	−
*NAG*	−	−	−	−	−	+
*MAL*	−	−	−	−	−	+
*GNT*	+	−	−	−	−	−
*MLT*	+	−	−	−	−	+
*OX*	+	+	+	+	+	+
***Growth***						
*37*°*C*	+	+	−	−	−	−
*30*°*C*	+	+	+	−	+	−
*23*°*C*	+	+	+	+	+	+
*4*°*C*	−	+	−	−	−	+
*0%NaCl*	+	+	+	w	+	+
*3% NaCl*	+	+	+	+	+	+
*6% NaCl*	+	+	+	+	+	+
*10% NaCl*	−	+	−	−	−	+
***Phenotype***						
*Hemolysis*	+	−	−	NG	−	−
*Catalase*	−	−	+	−	+	−

*Vibrio parahaemolyticus (Vp), Vibrio litoralis (Vl), Vibrio scophthalmi (Vs), Vibrio gallaecicus (Vg), Aliivibrio fischeri (Af) and Vibrio hibernica (Vh). Positive result (+), negative result (−), weak positive result (w), no growth (NG), underlined represents fermentation.*

The growth temperature range was different for each of the species, with *V. litoralis* (Vl) growing at all of the temperatures tested (Table 3). *V. scophthalmi* (Vs), *V. gallaecicus* (Vg), and *A. fischeri* (Af) showed a limited temperature growth range. *V. hibernica* (Vh) demonstrated a preference for the lower temperatures and grew at 4°C after 16 h. The *V. hibernica* (Vh) strain did not grow at the higher temperatures tested unlike the mesophilic pathogen *V. parahaemolyticus* (Vp). All of the *Vibrio* strains grew with the addition of 6% NaCl, both *V. litoralis* (Vl) and the *V. hibernica* (Vh) strains showed a higher degree of salt tolerance, growing in the presence of 10% added NaCl (Table 3). None of the strains tested were halotolerant to 20% added NaCl. *V. hibernica* (Vh) did not demonstrate the ability to lyse erythrocytes (β-hemolysis). *V. scophthalmi* (Vs) and *A. fischeri* (Af) were both catalase positive in contrast to the brine isolate as well as the other tested strains.

To test the Wiltshire brine isolate’s practical capabilities for carbohydrate metabolism, an API CH50 kit was utilized (Table 4). We assayed *V. parahaemolyticus* (Vp) and *V. litoralis* (Vl) for comparative purposes. The other strains used in previous tests could not grow in the media used. Numerous conditions were tested to optimize the assay for growth of these strains; however, they were not culturable under the conditions specified by the manufacturers. This kit confirmed that *V. hibernica* (Vh) was able to metabolize glucose, malate, maltose, galactose, fructose, sucrose and contained the enzyme β-galactosidase. Genes annotated for the metabolism of these carbohydrates were present in the genome. *V. hibernica* showed the ability to metabolize D-ribose as noted in the genome annotation and this is an important meat flavor compound. Glycerol was metabolized by *V. hibernica* at 23°C weakly, however it was strongly metabolized at the lower temperature of 4°C. Genes for other sugar alcohols (D-arabitol and mannitol) are also annotated within the genome and both were utilized by *V. hibernica*. Strikingly, the *V. hibernica* isolated from brine was the only *Vibrio* tested that maintained (or improved) its ability to metabolize flavor development and industrially relevant compounds at 4°C (Table 4). It is important to note that the genome annotation corresponded with the phenotypic profile of *V. hibernica*. This demonstrates the usefulness of coupling genomic sequencing and annotation with phenotypic characterization, specifically with regards organoleptic qualities.

**TABLE 4:** *Vibrio* species carbohydrates metabolism patterns on an API 50 CH kit grown at 23°C and 4°C.

	23°C			4°C	
		
	*Vp*	*Vl*	*Vh*	*Vl*	*Vh*
***Test***					
*GLY*	−	−	w	−	+
*LARA*	+	+	−	w	−
*RIB*	−	−	+	−	+
*DXYL*	−	+	−	w	−
*GAL*	−	+	+	−	+
*GLU*	+	+	+	−	+
*FRU*	+	+	+	−	+
*MNE*	+	+	+	w	+
*MAN*	+	−	+	−	+
*NAG*	−	+	+	−	+
*MAL*	+	+	+	−	+
*SAC*	−	−	w	−	W
*TRE*	w	−	−	−	−
*AMD*	w	−	−	−	−
*GLYG*	w	−	−	−	−
*DARL*	−	−	+	−	+
*GNT*	+	w	−	w	−

*Vibrio parahaemolyticus (Vp), Vibrio litoralis (Vl), and Vibrio hibernica (Vh). Positive result (+) and negative result (−) and weak positive result (w).*

## Discussion

The application of bioinformatic (Tax4Fun) and molecular (NGS) tools to food processing related microorganisms is vital for the modernization, standardization, improved safety and optimization of traditional food processes (He et al., 2017; Behera et al., 2018; Woods et al., 2019). One of the key aspects of the traditional food process of Wiltshire curing is the role of the microbial community (Gardner, 1971). There is a compelling need to investigate the function and mechanism of action of the microbial community within Wiltshire brine and particularly how the microbiota contributes to the organoleptic qualities of the final product. This traditional food process maintains its microflora by cycling the brine. Spent brine is replenished with fresh brine in a 50:50 ratio. This brine is then used for the curing (5–7 days) of meat until the brine is defined as spent by an expert technician. This is roughly after approximately 40 days of use, after which the cycle begins anew. To investigate this process, we sampled brine at an initial timepoint (Day 0) which was immediately after replenishing the Wiltshire brine with half fresh cure. The second timepoint (Day 20) was when the brine was in full use and by Day 40 it was toward the spent phase of the brine. Bioinformatic functional community prediction software was applied to the microbiome data sets of three curing containers and tested at the three timepoints. Similar functional profiles were seen within the Day 0 and Day 20 replicates, however, on Day 40 the functionality of microbial community showed greater variability. The food industry strives to maintain product consistency. A functionally stable microbiome is crucial for food processing standardization and this variability highlights the necessity to replenish the brine at Day 40.

Within the two defined clusters (I and II) by the functional prediction analysis, there is a switch between functions from Day 0 to Day 20. Functions such as valine, leucine, and isoleucine biosynthesis which are initially highly enriched, were selected against by Day 20. Functions such as valine, leucine, and isoleucine degradation are enriched for by Day 20. A similar pattern is seen with lysine degradation and biosynthesis. There were a number of systems identified as relating to the metabolism of amino acids. Metabolic functions such as amino acid degradation and synthesis are valuable for the meat curing as they contribute to flavor development (Kirimura et al., 1969; Nishimura and Kato, 1988; Montel et al., 1998). A number of functions have been identified that are enriched for by Day 20, many of which have important flavor enhancing roles. These include the metabolism of amino acids, such as valine, leucine, and isoleucine degradation, cysteine and methionine metabolism and alanine, aspartate and glutamate metabolism (Solms, 1969; Gonda et al., 2010; Liu et al., 2012). Generally complex sugars do not give a sensory perception in humans, therefore the metabolism of complex sugars is an important function facilitating a positive flavor sensory response (Sclafani, 1987; Beauchamp, 2016). The enrichment of genes for the degradation of polysaccharides is highest at Day 20 and this would produce more sensory perceptive simple sugars. Both sugars and amino acids need to be transported into the cell for metabolism and ABC transporters often facilitate this process. It is clear in the functional enrichment, that ABC transporter are also increased by Day 20 (Hosie and Poole, 2001; Schneider, 2001). The use of Tax4Fun not only gives an insight into the potential functional activity within a Wiltshire brine over time but can also be used to provide a functional standard fully optimized signature for an active Wiltshire cure. While Tax4Fun offers an insight into potential metabolic capabilities of a system, follow-on functional studies would be needed, such as expression studies and metabolomics, to fully understand the function of microbial communities in brine.

We have demonstrated that *V. hibernica* is an integral part of an active Wiltshire brine microbiome. This bacterium differs from other genera found in the core microbial community, as it is not a Lactic Acid Bacteria (LAB). However, the metabolic potential of its genome clearly indicates involvement in organoleptic quality development. The *V. hibernica* isolated from Wiltshire brine demonstrated a unique phenotypic profile when compared to the other tested *Vibrio* species. The species was named due to its country of origin, Ireland. The reconstruction of the phylogenetic history of the *Vibrio* species was far better supported in the HK tree compared to the 16S rRNA gene tree and this result is further strengthened by other studies with similar outcomes (Tanaka et al., 2017). Phylogenic analysis of this species was seen to be distinct from its closest relatives, *V. rumoiensis* and *V. litoralis*. Both of these species as well as *V. algivorus*, *V. casei* and *V. aphrogenes* belong to the Rumoiensis clade and we propose *V. hibernica* be included into this clade (Tanaka et al., 2017). Two of the *in silico* methods of species definition identified *V. hibernica* as a novel species. Average nucleotide identity (OrthoANIu) is an improved algorithm of ANI which uses USEARCH as a fast clustering method. ANI has a species boundary cut-off of 95% similarity (Richter and Rossello-Mora, 2009; Tindall et al., 2010; Kim et al., 2014; Yoon et al., 2017). The other method used in this study was *in silico* DNA-DNA Hybridization (DDH), where the same species cannot differ in the G + C content by more than 1% by the Genome-To-Genome Distance Calculator (GGDC) pairwise comparison (Meier-Kolthoff et al., 2014). When compared to the other *Vibrio* genomes, *V. hibernica* showed less than 95% ANI and greater than 1% G + C difference by *in silico* DDH, thus supporting this bacterium as a new species.

Flavor is one of the most important components of food products and gaining knowledge into the contributions of microbial genomics to flavor and other organoleptic properties is vital. This is especially the case for the advancement and understanding of traditional meat processes such as Wiltshire curing. The annotated *V. hibernica* genome has a number of genes that could aid in the development of the specific organoleptic properties of Wiltshire cured hams. Many genes in the genome of *V. hibernica* were annotated as being involved in the biosynthesis and degradation of amino acids and these have an important role in flavor development, specifically with the production of volatile flavor compounds (Nishimura and Kato, 1988). The enzymes involved in the degradation of the essential amino acid methionine are present in the genome, and this degradation pathway is involved in the production of important aroma compounds, particularly volatile sulfur compounds which are associated with desirable flavors in cured meats (Ferchichi et al., 1985; Weimer et al., 1999; Pripis-Nicolau et al., 2004). Other amino acids such as serine and cysteine have an important role in flavor. Serine has previously been reported as having a sweet and umami taste (Kirimura et al., 1969). Cysteine along with ribose can undergo the Maillard reaction, yielding a typical meat-like flavor (Mottram, 1998). The degradation of RNA is also a key component to flavor development and the genome has the genetic potential to do this. Nucleic acid degradation can produce 5′-guanosine monophosphate (5′-GMP) (Nagodawithana et al., 2013). This compound is an important flavor enhancer (Kuninaka, 1960; Ikeda, 2002; Ninomiya, 2015).

The breaking down of complex sugars into more simple sugars is a vital function in the curing/fermenting of Wiltshire cured hams, as there is a consumer preference for di- and mono-saccharide over more complex carbohydrates (Sclafani, 1987). The metabolism of lipids, fatty acid and isoprenoids into smaller compounds also leads to a value-added product with an increased preference for taste, accompanied by an increase in umami and kokumi tastes in a variety of products (Gänzle, 2009; Feng et al., 2014). Genes encoding the osmolyte betaine are also present in the genome. Osmolytes may confer the high salt tolerance of *V. hibernica*. Moreover, betaine is known to have an enhancing effect on taste receptors responses and improving intestinal function (Kiyohara et al., 1994; Wang et al., 2018). Additional benefits of increased dietary betaine levels have been linked to health benefits, specifically with decreasing the risk of developing several severe diseases (McGregor et al., 2002; Ross et al., 2011; Ying et al., 2013). It is important to note that *V. hibernica* was capable of growing and metabolizing sugars at 4°C, fermenting meat products and improving the organoleptic properties at this temperature is highly advantageous to the food industry as most detrimental bacteria are mesophilic organisms. Fermentation at lower temperatures though often comes with difficulties such as slow bacterial growth, extended lag phases, decreased industrial processing and increase financial costs (Uchimoto and Cruess, 1952; Charoenchai et al., 1998; Llauradó et al., 2002; Chiva et al., 2012). Thus, *V. hibernica* and its ability to metabolize sugars at low temperatures would be highly advantageous to a variety of industrial applications.

Another important characteristic of a Wiltshire cured product is the maintenance and depth of color of cured meat products, specifically with regards to the red and iridescent appearance of hams. The machinery for the production of heme is present in the genome of the *V. hibernica* isolate and this plays a vital role in color development (Chasco et al., 1996; Hugas and Monfort, 1997; Krause et al., 2011). Moreover, heme is currently being added to vegetarian food to produce meat flavored products (Fraser et al., 2018). An important parameter of Wiltshire curing brine is that nitrate reducing bacteria are promoted and maintained in the microbial milieu. The sequenced genome has 39 genes related to nitrate metabolism and the metabolism of nitrates also contributes to the development of the distinct red color of cured meats (Krause et al., 2011).

As well as evidence of genes enhancing organoleptic properties, a number of sequences were annotated as being involved in the production of vitamins and coenzymes. The potential increase in these during the curing process could yield a market for the cured meat product as a value added nutraceutical (Malik, 2007). There are a number of genes present for the biosynthesis of vitamins, such as B_1_, B_2_, B_7_, B_9_, B_12_ and K_2_, as well as antioxidants such as ubiquinone and glutathione. Vitamin B_1_ is an essential cofactor generally produced by plants, but never by vertebrates (Takusagawa et al., 1998; Rodionov et al., 2002). Vitamins B_2_ and B_7_ have important functions with regards to cellular processes (Powers, 2003; Heidker et al., 2016). Vitamin B_9_ is an essential vitamin necessary for human health and development (Ebara, 2017; Naderi and House, 2018). Vitamin K_2_ has positive effects on preventing osteoporosis and cardiovascular disease, as well as aiding in blood coagulation (Mahdinia et al., 2017). Studies have already been employed to produce vitamin K_2_ enriched products by traditional fermentation food processes (Mahanama et al., 2011; Singh et al., 2015). The vitamin B_12_ is an important cofactor for many enzymes (Cheng et al., 2016; Bridwell-Rabb and Drennan, 2017). Moreover this compound is known for its deep red color, which is a vital characteristic of Wiltshire cured products (Scott et al., 1964). Ubiquinone and glutathione are known for their antioxidant properties (Wang and Hekimi, 2016). Additionally glutathione is known to contribute to the kokumi flavor of a product (Tang et al., 2017). In a world where there is a trend for a decrease in excessive meat consumption, optimizing and increasing the nutritional and vitamin content of the meat is imperative, moreover curing meat has previously been shown to increase the nutritional value of the product (Okoń et al., 2017; Neff et al., 2018). Thus, the manipulation of a microbiome and the individual microbial components to use their full genetic potential would be beneficial to a number of industries. The absence of pathogenicity genes is a vital trait, if the bacteria is to be used in the food industry.

## Conclusion

The curing of ham in brine is a traditional artisan process that has been widely utilized in food processing for many years. The curing of meat often has an associated natural microbial diversity that can enhance the quality of the final product. We have investigated an important traditional value-add food process, Wiltshire curing, and delved into the microbial milieu that are an important part of this process. Molecular techniques were coupled with NGS to confirm the persistence of the core microbiome previously identified (Woods et al., 2019). We completed a predicted functional analysis of a fully compliant brine and identified functions that are enriched during each stage in the life cycle of the curing process. A *Vibrio* species has previously been proven to positively affect the organoleptic qualities in Wiltshire cured hams (Petaja et al., 1972). A new *Vibrio* isolate was cultured from an active Wiltshire brine with distinct phenotypic characteristics. Moreover, the food associated microbe was phylogenetically and genetically distinct from other published *Vibrio* species. We have defined this species as *Vibrio hibernica* strain B1.19, due to its isolation on the island of Ireland. We examined the genome of *V. hibernica* and highlighted several processes and genes that may contribute to the distinct specific organoleptic qualities of the Wiltshire product. The contribution of the enzymatic action of bacteria in food technology requires further investigation to evaluate the exact process behind this complex science. Our study gives an insight into the value-add processing of meat by a bacterium in a Wiltshire compliant fully functional brine.

## Data Availability Statement

The raw reads of the Microbiome dataset are publicly available in the Sequence Read Archive (SRA) of the NCBI Database under Accession numbers; SRR11073122, SRR11073123, SRR11073124, SRR11073125, SRR11073126, SRR11073127, SRR11073128, SRR11073129, SRR11073130, SRR11073131, SRR11073132, SRR11073133, SRR11073134, SRR11073135, SRR11073136, SRR11073137, SRR11073138, SRR11073139, SRR11073140, SRR11073141, SRR11073142, SRR11073143, SRR11073144, SRR11073145, SRR11073146, SRR11073147, and SRR11073148. The data of the genome sequence of *V. hibernica* that is studied here is deposited in DDBJ/ENA/GenBank under the accession VHKO00000000.

## Author Contributions

DW, IK, and FO’G were involved in the conception and authorship of the work. DW and IK both contributed to the experimental work.

## Conflict of Interest

The authors declare that the research was conducted in the absence of any commercial or financial relationships that could be construed as a potential conflict of interest.

## Footnotes

1 http://tax4fun.gobics.de/

2 www.eurofinsgenomics.eu

3 https://www.ezbiocloud.net/tools/ani

4 http://ggdc.dsmz.de/ggdc.php

5 http://www.ncbi.nlm.nih.gov/

## Supplementary Material

FIGURE S1 Microbiome characterization of an active Wiltshire brine, sampled from three monitored brine containers at three time point (Day 0, Day 20, and Day 40) in late 2016. The genera are represented as a relative percentage of the sample’s total microbiome. The dominant genera present are *Marinilactibacillus, Carnobacterium, Leuconostoc, Vibrio, Photobacterium* and genera present in <1% (Other).

FIGURE S2 Phylogenetic tree of the 16S rRNA genes from selected *Vibrio* species. The evolutionary history was inferred by using the Maximum Likelihood method based on the Kimura 2-parameter model. A discrete Gamma (+G) distribution was used with rate variation model to allow evolutionarily invariable (+I). The phylogeny was tested using 1,000 Bootstrap Replicates. Three outgroups were used to root the tree: *Salinivibrio costicola*, *Enterovibrio coralii*, and *Photobacterium lutimaris*. There were a total of 1212 positions in the final dataset. *Vibrio hibernica* is indicated by a black triangle.

TABLE S1 Accession numbers for the strains used for the reconstruction of the 16S rRNA phylogenetic tree.

TABLE S2 Accession numbers for the strains used for the reconstruction of the HK phylogenetic tree.
