# Supplementary figures and images for: Microbiome and Functional Analysis of a Traditional Food Process: Isolation of a Novel Species (Vibrio hibernica) With Industrial Potential
Source: Front Microbiol. 2020 Apr 9;11:647. doi: 10.3389/fmicb.2020.00647 (PMC7179675; doi:10.3389/fmicb.2020.00647)

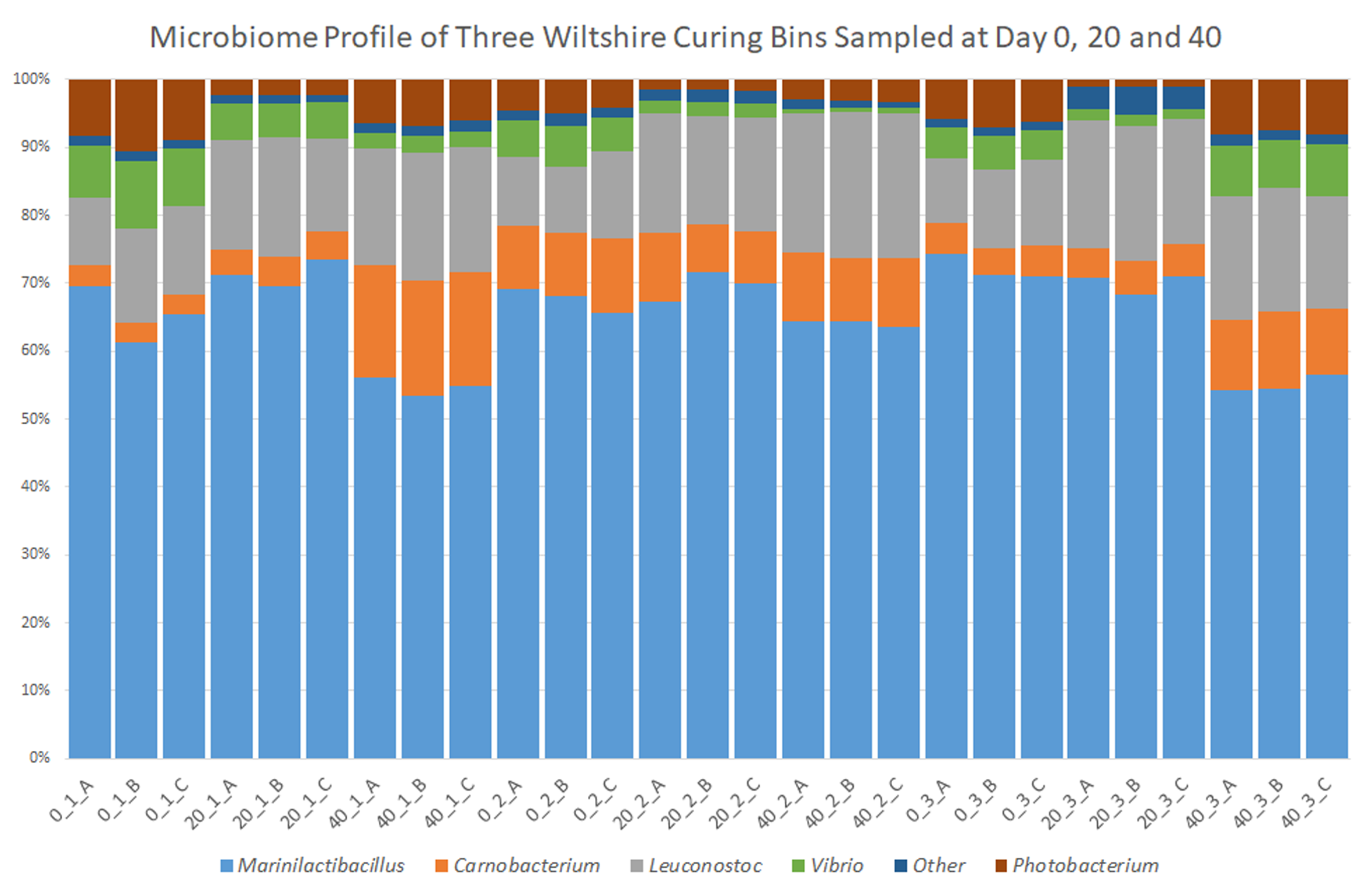

Supplement: FIGURE S1 — Microbiome characterization of an active Wiltshire brine, sampled from three monitored brine containers at three time point (Day 0, Day 20, and Day 40) in late 2016. The genera are represented as a relative percentage of the sample’s total microbiome. The dominant genera present are Marinilactibacillus, Carnobacterium, Leuconostoc, Vibrio, Photobacterium and genera present in <1% (Other). [file Image_1.TIF]

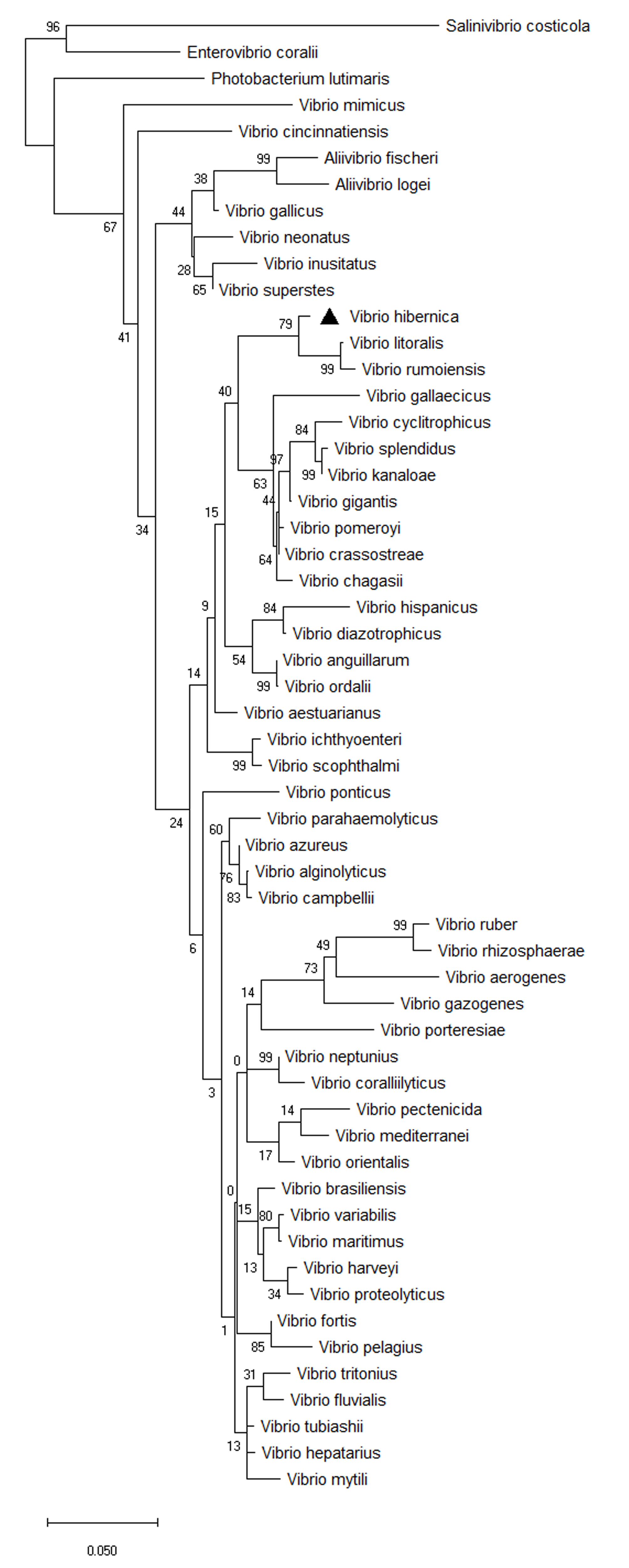

Supplement: FIGURE S2 — Phylogenetic tree of the 16S rRNA genes from selected Vibrio species. The evolutionary history was inferred by using the Maximum Likelihood method based on the Kimura 2-parameter model. A discrete Gamma (+G) distribution was used with rate variation model to allow evolutionarily invariable (+I). The phylogeny was tested using 1,000 Bootstrap Replicates. Three outgroups were used to root the tree: Salinivibrio costicola, Enterovibrio coralii, and Photobacterium lutimaris. There were a total of 1212 positions in the final dataset. Vibrio hibernica is indicated by a black triangle. [file Image_2.TIF]
