# Supplementary material for: Microbiome and Functional Analysis of a Traditional Food Process: Isolation of a Novel Species (Vibrio hibernica) With Industrial Potential
Source: Front Microbiol. 2020 Apr 9;11:647. doi: 10.3389/fmicb.2020.00647 (PMC7179675; doi:10.3389/fmicb.2020.00647)
Supplement: Supplementary file 5 [file Data_Sheet_1.docx]

Supplementary Material

## Supplementary Figures

**Figure S1:** Microbiome profiles of three Wiltshire curing bins sampled in triplicate on day 0, 20 and 40 in late 2016. Bacterial genera are represented as a relative percentage of the sample’s total microbiome. Letters A, B and C correspond to sample replicates. The microbiome consists of *Marinilactibacillus*, *Carnobacterium*, *Leuconostoc*, *Vibrio*, *Photobacterium* and “Other”. The “Other” group consisted of relative abundances of less than 1% individually.

**Figure S2:** Phylogenetic tree of 16S rRNA genes from selected *Vibrio* species. The evolutionary history was inferred by using the Maximum Likelihood method based on the Kimura 2-parameter model. A discrete Gamma (+G) distribution was used with rate variation model to allow evolutionarily invariable (+I). The phylogeny was tested using 1,000 Bootstrap Replicates. Three outgroups were used to root the three: *Salinivibrio costicola*, *Enterovibrio coralii* and *Photobacterium lutimaris*. There were a total of 1212 positions in the final dataset. *Vibrio hibernica* is indicated by a black triangle.

**
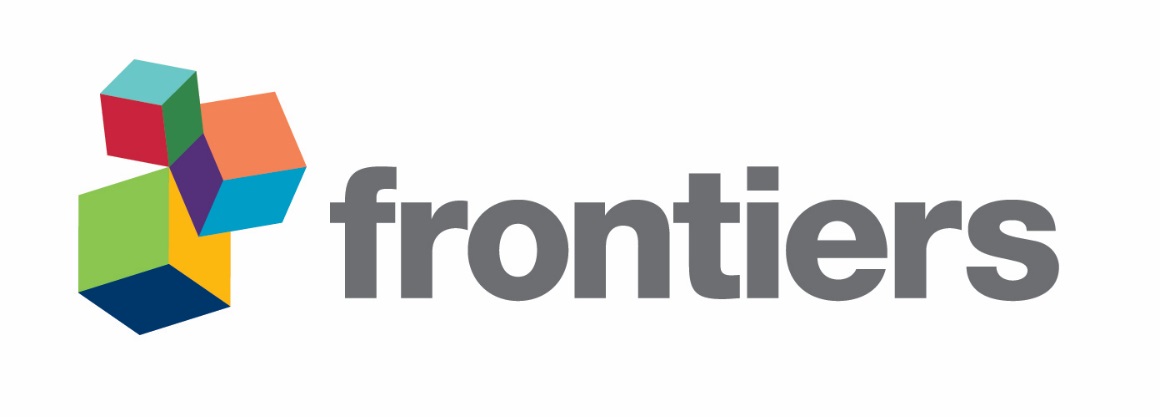
**
